# Clinical, Pathological, and Antimicrobial Characteristics of *Pasteurella multocida* Infections in Extensively Reared Rabbits in Western Romania

**DOI:** 10.3390/vetsci13050485

**Published:** 2026-05-17

**Authors:** Vlad Iorgoni, Livia Stanga, Paula Nistor, Alexandru Gligor, Janos Degi, Bogdan Florea, Gabriel Orghici, Ionica Iancu, Cosmin Horatiu Maris, Ioan Cristian Dreghiciu, Viorel Herman

**Affiliations:** 1 Department of Infectious Diseases and Preventive Medicine, Faculty of Veterinary Medicine, University of Life Sciences “King Mihai I” from Timişoara, 300645 Timisoara, Romania; vlad.iorgoni@usvt.ro (V.I.); paula.nistor@usvt.ro (P.N.); alexandru.gligor@usvt.ro (A.G.); janosdegi@usvt.ro (J.D.); gabriel.orghici@usvt.ro (G.O.); ionica.iancu@usvt.ro (I.I.); viorel.herman@fmvt.ro (V.H.); 2 Doctoral School “Veterinary Medicine”, University of Life Sciences “King Mihai I” from Timişoara, Calea Aradului 119, 300645 Timisoara, Romania; bogdan-alexandru.florea.fmv@usvt.ro; 3 Discipline of Microbiology, Faculty of Medicine, “Victor Babes” University of Medicine and Pharmacy, Eftimie Murgu Square 2, 300041 Timisoara, Romania; 4 Department of Internal Medicine, University of Life Sciences “King Mihai I” from Timişoara, 300645 Timisoara, Romania; 5 Department of Forestry, Faculty of Engineering and Applied Technologies, University of Life Sciences “King Mihai I” from Timișoara, 300645 Timisoara, Romania; cosmin.maris@usvt.ro; 6 Discipline of Parasitology, Faculty of Veterinary Medicine, University of Life Sciences “King Mihai I” from Timișoara, 300645 Timisoara, Romania; cristian.dreghiciu@usvt.ro; 7 Academy of Romanian Scientists (AOSR), Str. Ilfov, Nr. 3, Sector 5, 50044 Bucharest, Romania

**Keywords:** rabbit, pasteurellosis, *Pasteurella multocida*, antimicrobial resistance, respiratory disease, traditional farming

## Abstract

Pasteurellosis is a major bacterial disease of domestic rabbits, commonly associated with respiratory disorders, abscesses, reproductive pathology, and systemic infections. This study investigated the occurrence, clinical manifestations, pathological lesions, and antimicrobial susceptibility of bacterial isolates obtained from rabbits raised in traditional extensive systems in western Romania, with identification of *Pasteurella multocida* performed using matrix-assisted laser desorption/ionization time-of-flight mass spectrometry (MALDI-TOF MS). A total of 308 rabbits from 23 holdings were clinically examined, necropsied when applicable, and subjected to bacteriological analysis. Clinical signs compatible with pasteurellosis were observed in 132 rabbits (42.9%), including respiratory diseases, abscess formation, otitis, and reproductive disorders. Samples collected from affected and deceased rabbits were cultured and analyzed using MALDI-TOF MS, confirming 87 isolates as *P. multocida*. Antimicrobial susceptibility testing using the VITEK 2 system revealed high resistance to tetracyclines (63.22%) and beta-lactams (55.17%), while higher susceptibility was observed for enrofloxacin (91.95%), gentamicin (89.66%), ciprofloxacin (86.21%), and florfenicol (80.46%). The presence of multidrug-resistant isolates highlights the need for laboratory-guided antimicrobial therapy and improved biosecurity measures in traditional rabbit holdings.

## Simple Summary

Pasteurellosis is a major infectious disease of domestic rabbits, causing respiratory and systemic infections associated with significant economic losses. The disease is mainly caused by Pasteurellosis and the opportunistic bacterium *Pasteurella multocida*, which may persist in healthy carriers and become pathogenic under stress or poor environmental conditions. This study investigated pasteurellosis in rabbits from traditional holdings in western Romania, focusing on epidemiology, clinical signs, pathological lesions, and antimicrobial resistance. A total of 308 rabbits were examined, of which 132 showed clinical signs consistent with pasteurellosis. Eighty-seven isolates were identified as *P. multocida* using MALDI-TOF MS with high-confidence scores (≥2.0). Antimicrobial susceptibility testing using the VITEK 2 system revealed high resistance to tetracyclines and beta-lactams, whereas fluoroquinolones, aminoglycosides, and florfenicol showed higher efficacy. The findings emphasize the importance of laboratory-based diagnosis and targeted antimicrobial therapy in rabbit pasteurellosis management.

## 1. Introduction

Pasteurellosis is widely recognized as a major infectious disease in domestic rabbits, causing significant morbidity, mortality, and economic losses in both intensive and extensive production systems [1,2,3]. It is primarily caused by *Pasteurella multocida*, a Gram-negative, non-motile coccobacillus that colonizes the upper respiratory tract and may persist in clinically healthy animals as part of the commensal microbiota [1,2,3]. This asymptomatic carrier state represents a key epidemiological factor, facilitating silent pathogen dissemination and long-term maintenance within populations. Under stress or immunosuppression, *P. multocida* can shift from a commensal organism to an opportunistic pathogen, leading to a broad spectrum of clinical manifestations [1,2,3,4,5].

The clinical expression of pasteurellosis in rabbits is heterogeneous and can affect multiple organ systems. Respiratory involvement is the most common form, typically presenting as chronic rhinitis with sneezing, mucopurulent nasal discharge, and conjunctivitis. However, the infection may extend beyond the respiratory tract, causing subcutaneous abscesses, otitis media and interna, mastitis, and reproductive disorders such as metritis and pyometra. In severe cases, systemic spread can lead to septicemia and death [1,4,5,6]. Disease pathogenesis is closely linked to key virulence factors of *Pasteurella multocida*, including capsule formation, endotoxin production, and adhesion mechanisms that promote colonization, immune evasion, and tissue invasion [1,4,6,7,8,9].

Environmental and management-related factors play a fundamental role in the development and progression of pasteurellosis. In traditional extensive rabbit farming systems, animals are often exposed to suboptimal conditions, including poor ventilation, high humidity, temperature fluctuations, and overcrowding, all of which contribute to stress and increased susceptibility to infection. Furthermore, the frequent introduction of new animals without appropriate quarantine measures, as well as the participation in exhibitions and animal exchanges, may facilitate the spread of the pathogen between different holdings [6,10,11,12]. These conditions create a favorable environment for the persistence and transmission of *P. multocida*, leading to recurrent outbreaks and chronic disease evolution [1,6,10,11,12]. Suboptimal housing conditions and environmental stressors have also been shown to negatively affect rabbit welfare, increasing susceptibility to infectious diseases and impairing productivity [13].

Another major concern associated with rabbit pasteurellosis is the increasing occurrence of antimicrobial resistance. The empirical use of antibiotics, often without prior microbiological confirmation or susceptibility testing, has contributed to the selection of resistant strains, reducing treatment efficacy and complicating disease control strategies [13,14,15,16,17]. This phenomenon is particularly relevant in traditional breeding systems, where access to advanced diagnostic tools may be limited and therapeutic decisions are frequently based on empirical protocols [14,15,16,17,18].

In recent years, significant advances in diagnostic microbiology have improved the ability to accurately identify bacterial pathogens and assess their antimicrobial susceptibility. Matrix-assisted laser desorption/ionization time-of-flight mass spectrometry has emerged as a rapid and reliable method for bacterial identification, allowing precise species-level discrimination based on protein spectral analysis. In parallel, automated antimicrobial susceptibility testing systems such as VITEK 2 have enhanced the standardization and reproducibility of resistance profiling, providing clinically relevant results in a timely manner [18,19,20,21,22,23]. The integration of these technologies into veterinary diagnostics represents a major step forward in the management of infectious diseases, enabling targeted therapy and supporting antimicrobial stewardship [1,19,20,21,22,23,24].

Despite the well-established significance of pasteurellosis, comprehensive data on its epidemiological distribution, clinical presentation, pathological features, and antimicrobial resistance patterns in rabbits raised under traditional extensive systems in Eastern Europe and Romania remain scarce. In this context, the present study aims to fill this gap by investigating the occurrence, clinical manifestations, and lesions associated with *Pasteurella multocida* infections in domestic rabbits from western Romania while also evaluating their antimicrobial susceptibility profiles. This study integrates field observations with advanced laboratory confirmation methods, including MALDI-TOF MS and VITEK 2, to provide a more accurate and contemporary characterization of the infection under traditional rearing conditions.

## 2. Materials and Methods

### 2.1. Study Area and Rabbit Holdings

This study was conducted in 23 traditional rabbit holdings located in western Romania, characterized by small-scale extensive breeding systems in which animals were raised under variable environmental conditions and with limited implementation of biosecurity measures. These holdings included both hobby breeders and small-scale producers, some of whom participated in rabbit exhibitions, thus increasing the risk of pathogen introduction and dissemination. Housing conditions varied between holdings but generally involved outdoor or semi-enclosed facilities, which are often associated with fluctuating temperature and humidity levels, as well as variable sanitation practices.

Ethical review and approval were waived for this study by the Bioethics Commission of the University of Life Sciences “King Mihai I” from Timișoara, Romania (No. 667/01.04.2026) on 1 April 2026, as the research did not involve the use of live animals in an experimental setting and consisted of clinical and diagnostic procedures performed under routine field conditions.

### 2.2. Animals Included in the Study

A total of 308 rabbits were included in the study, representing multiple breeds such as German Giant, German Spotted Giant, Rex, Blanc de Pannonia, German Lop, and dwarf breeds. The age of the animals ranged from approximately two months to three years and both males and females were included in the experiment. The studied population was heterogeneous in terms of physiological status, including growing, breeding, and lactating individuals, which allowed for a comprehensive assessment of disease occurrence across different categories.

All animals were systematically examined clinically. Samples were collected from rabbits presenting clinical signs suggestive of pasteurellosis, as well as from animals that died during the study period.

### 2.3. Epidemiological and Clinical Examination

An epidemiological investigation was performed in each holding in order to assess the potential risk factors associated with the occurrence and spread of pasteurellosis. Data were collected regarding housing conditions, animal density, feeding practices, introduction of new animals, and general management procedures. Particular attention was given to identifying conditions that could contribute to stress or immunosuppression.

All animals underwent a thorough clinical examination, focusing on the identification of signs consistent with pasteurellosis. The examination included evaluation of the respiratory system, skin and subcutaneous tissues, mammary glands, reproductive tract, and neurological status. Clinical signs such as sneezing, nasal discharge, ocular discharge, abscess formation, otitis, and reproductive abnormalities were recorded and analyzed.

Seasonal data were recorded during field investigations and categorized according to the period of occurrence of clinical cases.

### 2.4. Sampling

Samples were collected from both animals exhibiting clinical signs suggestive of pasteurellosis and individuals that died during the study period. In several cases, multiple samples were obtained from the same animal, resulting in a total of 286 collected specimens. These comprised 98 nasal swabs, 64 conjunctival swabs, 47 samples of abscess content, 39 lung tissue samples, and 38 internal organ samples collected during necropsy.

All samples were obtained under strict aseptic conditions and were promptly transported to the laboratory for bacteriological analysis to ensure sample integrity and reliability of the microbiological results.

### 2.5. Bacteriological Examination

Samples were cultured on Brain Heart Infusion agar and 5% sheep blood agar and incubated at 37 °C for 24 to 48 h under aerobic conditions. Colonies suspected of belonging to *Pasteurella* spp. were selected based on morphological characteristics and subjected to further identification using matrix-assisted laser desorption/ionization time-of-flight mass spectrometry [18,19,20,21]. The MALDI-TOF MS analysis was performed according to standard protocols, and identification was achieved by comparing the obtained spectra with reference databases [18,19,20]. Only identifications with score values ≥ 2.0 were considered reliable for species-level identification [19,20,21,22,25].

### 2.6. Antimicrobial Susceptibility Testing

Antimicrobial susceptibility testing was performed using the VITEK 2 automated system, which allows standardized evaluation of bacterial growth in the presence of predefined concentrations of antimicrobial agents. The system provided susceptibility profiles for multiple antibiotic classes, and results were interpreted according to current EUCAST guidelines [26,27,28,29,30]. The automated nature of the system ensured reproducibility and minimized subjective interpretation, providing reliable data regarding resistance patterns [28,29,30].

Multidrug resistance (MDR) was defined as resistance to at least one agent in three or more antimicrobial classes [25,26,27].

### 2.7. Statistical Analysis

This study was designed as an observational cross-sectional investigation. Descriptive statistics were used to summarize epidemiological, clinical, and microbiological data. Prevalence was calculated as the proportion of animals presenting clinical signs compatible with pasteurellosis among the total examined population, and results are presented with 95% confidence intervals (CIs).

Comparisons between groups were performed using chi-square tests, with the significance threshold set at *p* < 0.05. Statistical analyses were conducted using standard statistical software (GraphPad Prism version 10.0 Software, San Diego, CA, USA). Given the field-based nature of this study and the heterogeneity between holdings, the analyses were primarily exploratory.

## 3. Results

### 3.1. Epidemiological Findings

**Figure 1:**
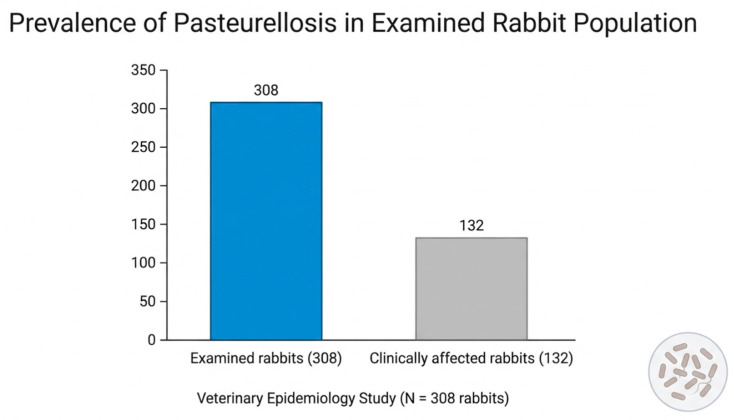
Distribution of rabbits presenting clinical signs compatible with pasteurellosis among the examined population (N = 308). A total of 132 rabbits presented clinical manifestations compatible with pasteurellosis.

Across the 23 investigated rabbit holdings, a total of 308 rabbits were clinically examined during the study period. Among these animals, 132 rabbits presented clinical signs compatible with pasteurellosis, corresponding to a proportion of 42.9% of rabbits (95% CI: 37.3–48.6). (Figure 1).

The occurrence of clinical cases varied between holdings but was observed in all investigated locations, indicating the widespread presence of the disease in the studied rabbit populations; from a total of 308 examined rabbits, 132 presented clinical signs.

### 3.2. Clinical Findings

Clinical examination revealed a wide range of manifestations consistent with pasteurellosis. The most frequently observed signs included sneezing, mucopurulent nasal discharge, ocular discharge, and subcutaneous abscess formation. In addition, several animals presented with otitis externa, torticollis, mammary abscesses, and reproductive tract disorders.

Subcutaneous abscesses represented one of the most common clinical findings and were typically located on the head, neck, and limbs. In several cases, these lesions showed a recurrent character, reappearing after surgical drainage. The abscesses usually contained dense, whitish purulent material.

Mammary abscesses were detected in females both during lactation and outside the lactation period, suggesting that infection may occur independently of lactation-associated trauma. In addition, a number of females developed pyometra, which was sometimes accompanied by purulent vaginal discharge, indicating the involvement of the reproductive tract in systemic infection.

**Figure 2:**
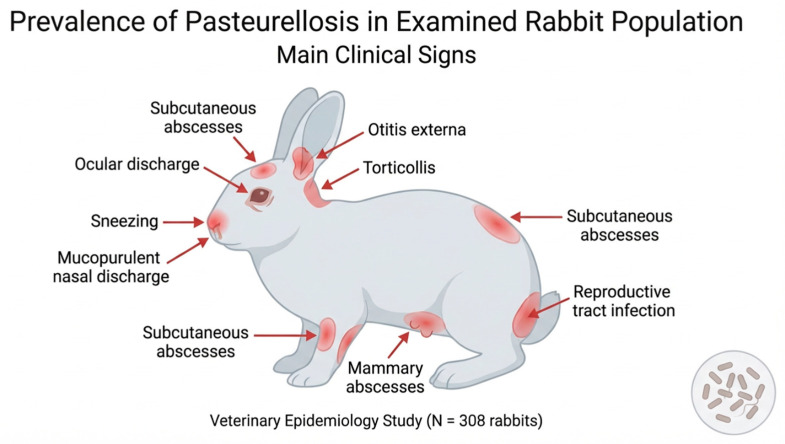
Distribution of the main clinical manifestations observed in rabbits affected by pasteurellosis.

The distribution of clinical manifestations observed in the affected rabbits is summarized in Figure 2 and Table 1.

### 3.3. Necropsy Findings

Necropsy examinations performed on 42 rabbits that died during the study revealed multiple pathological lesions affecting several organ systems. The most common thoracic lesions included pulmonary congestion, pleuritis, pulmonary abscesses, and pyothorax, indicating severe respiratory involvement.

Pulmonary abscesses varied considerably in size and, in some cases, occupied a large portion of the affected lung tissue. In the abdominal cavity, lesions such as peritonitis, hepatomegaly, splenomegaly, and uterine pathology were frequently recorded. The presence of fibrin deposits and adhesion receptors between abdominal organs suggested advanced inflammatory processes and possible septic dissemination.

**Figure 3:**
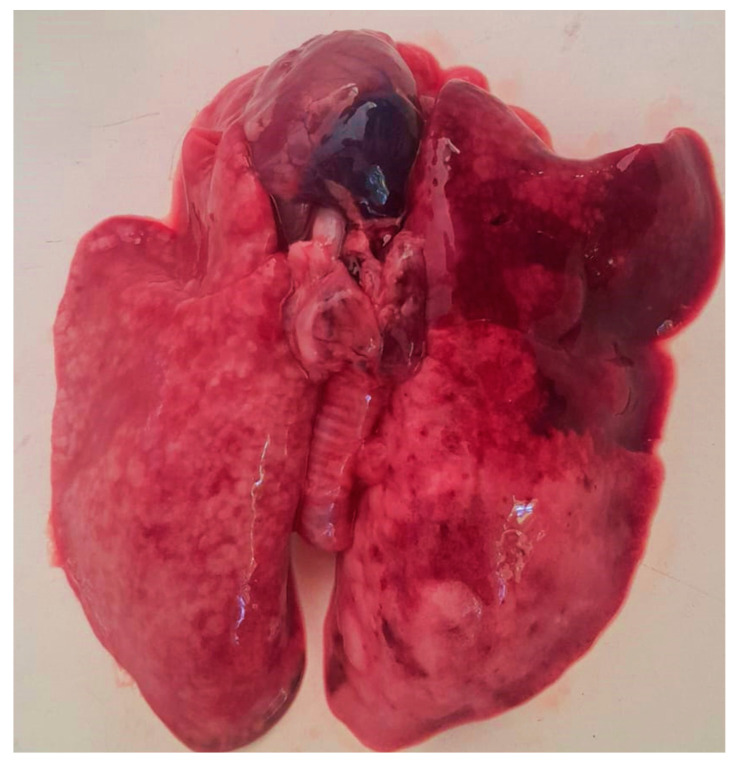
Representative necropsy lesions observed in rabbits with pasteurellosis. The lungs show diffuse congestion and multifocal coalescing areas of consolidation affecting multiple lobes bilaterally. The lesion pattern is consistent with severe respiratory involvement associated with Pasteurella multocida infection.

Representative pathological lesions observed during necropsy are illustrated in Figure 3.

### 3.4. Bacteriological Findings

Bacteriological analysis led to the recovery of 87 bacterial isolates, all of which were identified as *Pasteurella multocida* by MALDI-TOF mass spectrometry. Pure cultures were obtained on blood agar following subculture, with similar growth also observed on BHI agar. Colonies grown on 5% sheep blood agar appeared consistently grayish, smooth to slightly mucoid, and non-hemolytic, reflecting a uniform cultural profile. These phenotypic characteristics supported microbiological identification. Microscopic examination further revealed Gram-negative coccobacilli, frequently exhibiting bipolar staining, a hallmark feature of *Pasteurella multocida.* The overall uniformity of the phenotypic and proteomic findings reinforces the reliability of the identification results obtained through MALDI-TOF analysis (identification scores ≥ 2.0), confirming species-level identification of *P. multocida*.

### 3.5. Antimicrobial Susceptibility

**Figure 4:**
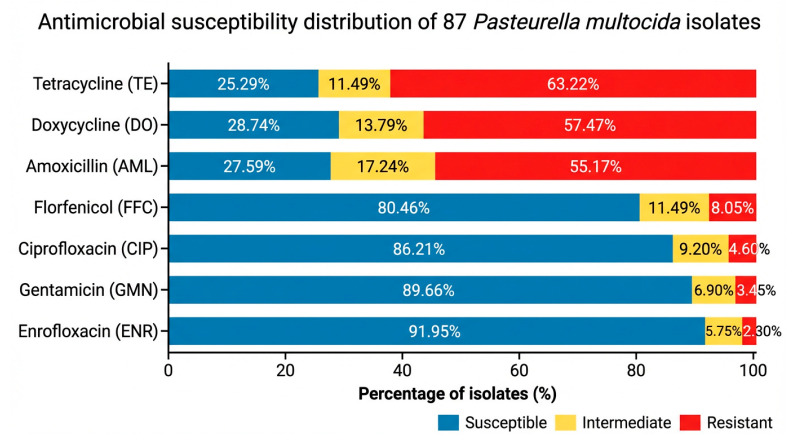
Antimicrobial susceptibility distribution of 87 *Pasteurella multocida* isolates recovered from domestic rabbits raised under traditional extensive systems in western Romania. Bars represent the proportion (%) of isolates classified as susceptible, intermediate (increased exposure), and resistant according to EUCAST-based interpretation. High resistance rates were observed for tetracycline (63.22%), doxycycline (57.47%), and amoxicillin (55.17%), whereas enrofloxacin (91.95%), gentamicin (89.66%), ciprofloxacin (86.21%), and florfenicol (80.46%) showed the highest susceptibility profiles. Exact percentages are indicated above each bar.

Antimicrobial susceptibility testing performed on the 87 *P. multocida* isolates revealed variable susceptibility patterns across the tested antimicrobial agents (Figure 4). High resistance levels were observed for tetracycline (63.22%) and doxycycline (57.47%), followed by amoxicillin (55.17%). In contrast, the highest susceptibility rates were recorded for enrofloxacin (91.95%), gentamicin (89.66%), ciprofloxacin (86.21%), and florfenicol (80.46%). Based on the MDR definition criteria, 39 isolates (44.8%) were classified as multidrug-resistant.

Overall, the antimicrobial susceptibility profile emphasize the importance of performing antimicrobial susceptibility testing prior to initiating treatment.

## 4. Discussion

The results of the present study confirm that pasteurellosis remains a major health concern in rabbits raised under traditional extensive systems, with a high proportion of rabbits presenting clinical signs compatible with this disease. The detection of clinical signs in 42.9% of the examined animals indicates a substantial burden of disease within the studied population and suggests that *Pasteurella multocida* is widely distributed in these holdings. The presence of infection in all investigated farms further supports the hypothesis of endemic circulation, likely maintained through asymptomatic carrier animals.

The diversity of clinical manifestations (respiratory signs, abscess formation, otitis, and reproductive disorders) observed in this study is consistent with the known polymorphic nature of pasteurellosis. Respiratory signs predominated, reflecting the primary localization of the pathogen in the upper respiratory tract, but the frequent occurrence of abscesses, otitis, and reproductive disorders highlights the capacity of *P. multocida* to disseminate and affect multiple organ systems [1,5,10,28]. The recurrent nature of abscesses observed in several cases may be explained by the particular characteristics of purulent material in rabbits, which tends to be dense and encapsulated, thereby limiting antibiotic penetration and complicating treatment [1,31,32,33,34].

Most clinical cases were recorded during cold and humid periods, particularly during the autumn season, suggesting that environmental conditions such as temperature fluctuations, increased humidity, and poor ventilation may have contributed to the onset or exacerbation of the disease.

The necropsy findings further emphasize the severity of the disease, revealing extensive lesions in both thoracic and abdominal cavities. The presence of bronchopneumonia, pleuritis, and pyothorax indicates advanced respiratory involvement, while abdominal lesions such as peritonitis and organomegaly suggest systemic dissemination. These findings are indicative of severe infection and may explain the mortality observed in affected animals. These findings are consistent with previous reports describing bronchopneumonia and systemic dissemination associated with *P. multocida* infections in rabbits [2,5,12,28].

The use of MALDI-TOF mass spectrometry for bacterial identification represents a significant methodological advantage of this study, allowing rapid and accurate identification of *P. multocida* isolates. Previous studies demonstrated that MALDI-TOF MS provides higher specificity and significantly shorter turnaround times compared to conventional biochemical methods, thereby improving diagnostic efficiency in veterinary microbiology laboratories [19,20,21,22]. In the present study, all isolates yielded identification scores ≥ 2.0, supporting the reliability of species-level identification. Similarly, the VITEK 2 automated system enabled standardized antimicrobial susceptibility testing with reduced operator-dependent variability and improved the reproducibility of results [28,29,30]. The integration of these diagnostic platforms facilitated rapid laboratory confirmation and supported a more accurate evaluation of antimicrobial resistance patterns among the recovered isolates.

A limitation of the present study is the absence of molecular epidemiological characterization of the isolates. Although MALDI-TOF MS ensured accurate species-level identification and VITEK 2 provided standardized antimicrobial susceptibility profiles, no typing approaches were applied to investigate the genetic relationships among isolates. Consequently, it remains unclear whether the recovered strains represent a shared circulating clone across holdings or multiple unrelated lineages with potentially different virulence and resistance backgrounds. Previous studies have demonstrated the utility of capsular and lipopolysaccharide genotyping, virulence-associated gene profiling, multilocus sequence typing (MLST), and whole-genome sequencing in elucidating the epidemiology and host adaptation of *Pasteurella multocida* [35,36,37,38,39]. The integration of such approaches in future investigations would provide a deeper understanding of transmission dynamics and improve the epidemiological interpretation of antimicrobial resistance patterns. The application of molecular epidemiological approaches such as multilocus sequence typing (MLST) and whole-genome sequencing (WGS) would also facilitate the investigation of transmission pathways between holdings, identification of dominant circulating clones, and characterization of virulence- and resistance-associated genetic determinants.

The recovery of 87 isolates in the present study reflects an extensive sampling strategy that included multiple types of specimens and, in some cases, repeated sampling from the same animal. This approach provides a more comprehensive overview of the distribution of *P. multocida* within affected populations and allows for a more accurate assessment of antimicrobial resistance patterns. The relatively high number of isolates also strengthens the statistical relevance of the findings.

The antimicrobial susceptibility results revealed a concerning level of resistance to commonly used antibiotics, particularly tetracyclines and beta-lactams. Based on this definition, some isolates were classified as multidrug-resistant, highlighting the clinical relevance of the resistance patterns observed in this study [25,26,27,40]. These findings are in line with previous reports and may be attributed to the widespread use of these antimicrobial agents in veterinary practice, often without prior susceptibility testing. The susceptibility profile observed in the present study is generally consistent with previous reports from rabbit and livestock-associated *Pasteurella multocida* isolates, which also described elevated resistance to tetracyclines and beta-lactams, while fluoroquinolones and florfenicol retained comparatively higher activity [16,17,41,42,43,44,45,46]. The higher susceptibility observed for fluoroquinolones, florfenicol, and aminoglycosides suggests potential therapeutic options; however, these findings must be interpreted with caution. In rabbit pasteurellosis, treatment outcomes are influenced not only by in vitro susceptibility results but also by the clinical presentation of the disease. Respiratory forms, abscess-forming infections, otitis, reproductive disorders, and septicemic cases differ substantially in terms of pathogenesis, tissue distribution, and response to therapy. In particular, abscesses in rabbits are characterized by dense, caseous material that limits antimicrobial penetration, often requiring surgical intervention or local management in addition to systemic treatment [1,5,12,39,40]. In this context, natural alternatives such as phytobiotics have been increasingly explored to reduce antibiotic use, with compounds like oregano demonstrating antimicrobial, antioxidant, and immunomodulatory effects in rabbits [41]. The high resistance rates observed may reflect cumulative antimicrobial exposure in traditional systems, where empirical treatments are frequently applied without prior susceptibility testing. Overall, the antimicrobial susceptibility profile emphasizes the importance of performing antimicrobial susceptibility testing prior to initiating treatment. These findings support the importance of selecting antimicrobial therapy based not only on in vitro susceptibility testing but also on the clinical presentation of the disease and prudent antimicrobial stewardship principles.

Furthermore, antimicrobial stewardship considerations should be taken into account, as favorable susceptibility profiles do not necessarily translate into effective bacterial eradication under field conditions. Experimental and field observations have shown that fluoroquinolone exposure, including enrofloxacin, may still select for reduced susceptibility over time. Therefore, antimicrobial selection should be guided by susceptibility testing, the clinical form of the disease, the route of administration, and prudent use principles, rather than relying solely on in vitro activity profiles [42,43,44,45,46].

The environmental and management factors identified during the epidemiological investigation likely played a key role in the occurrence and spread of the disease. Poor housing conditions, inadequate ventilation, and the lack of quarantine measures for newly introduced animals create an environment that facilitates pathogen transmission. Additionally, participation in exhibitions and animal exchanges may contribute to the dissemination of infections between holdings. These findings highlight the importance of implementing strict biosecurity measures and improving management practices in order to reduce the incidence of pasteurellosis [1,6,12,47,48].

From a One Health perspective, *P. multocida* also has zoonotic potential, as it is capable of causing infections in humans through animal contact. Therefore, improving biosecurity measures in rabbit holdings is essential not only for animal health but also for public health protection [5,9,12,49,50].

Overall, the results of this study underscore the importance of integrating advanced diagnostic methods into routine veterinary practice and emphasize the need for responsible antimicrobial use. The combination of accurate identification using MALDI-TOF MS and standardized susceptibility testing using VITEK 2 provides a reliable basis for targeted therapy and contributes to the control of antimicrobial resistance.

This study has several limitations that should be acknowledged. First, the sampling strategy was partially based on clinical presentation, which may have introduced selection bias and overestimated disease prevalence. Second, no control group of clinically healthy animals was included, limiting the ability to assess risk factors quantitatively. Third, the absence of molecular characterization restricts the epidemiological interpretation of the isolates. Finally, the lack of advanced statistical modeling limits the ability to establish causal relationships between management factors and disease occurrence. Despite these limitations, this study provides valuable field-based data from a region where such information remains scarce. Additionally, because the study was based on a cross-sectional observational design, causal relationships between the identified risk factors and disease occurrence could not be established.

## 5. Conclusions

The present study demonstrates a high proportion of rabbits presenting clinical manifestations compatible with pasteurellosis in rabbits raised under traditional conditions associated with diverse clinical and pathological manifestations. The detection of multidrug-resistant *Pasteurella multocida* strains highlights the importance of antimicrobial susceptibility testing prior to treatment. These findings support the implementation of targeted therapeutic strategies, improved biosecurity measures, and stricter control of antibiotic use in small-scale rabbit production systems.

## Figures and Tables

**Table 1:** Distribution of clinical findings in rabbits with pasteurellosis (n = 132).

Clinical Sign	Number of Rabbits (n)
Sneezing	84
Mucopurulent nasal discharge	91
Ocular discharge	76
Subcutaneous abscesses	68
Otitis externa	29
Torticollis	13
Mammary abscesses (females)	21
Reproductive tract disorders (total)	18
Pyometra	11
Purulent vaginal discharge	9

## Data Availability

The data presented in this study are available within the article. Further inquiries can be directed to the corresponding author.

## Abbreviations

The following abbreviations are used in this manuscript:

AML	Amoxicillin
BHI	Brain Heart Infusion
CIP	Ciprofloxacin
DO	Doxycycline
ENR	Enrofloxacin
EUCAST	European Committee on Antimicrobial Susceptibility Testing
FFC	Florfenicol
GMN	Gentamicin
R	Resistant
S	Susceptible
TE	Tetracycline
